# Patient-reported actions following receipt of pathogenic hereditary cancer genetic test results: results from a population-based screening study in primary care

**DOI:** 10.1186/s12875-026-03178-9

**Published:** 2026-02-18

**Authors:** Faith A. Beers, Tesla N. Theoryn, Emerson J. Dusic, Heather M. Harris, Sarah Knerr, DaLaina Cameron, Susan B. Trinidad, Barbara M. Norquist, Michael L. Raff, Jeannine M. Brant, Deborah J. Bowen, Elizabeth M. Swisher, Catharine Wang

**Affiliations:** 1 Department of Bioethics and Humanities, School of Medicine, University of Washington, Seattle, WA 98195 USA; 2 Department of Epidemiology, School of Public Health, University of Michigan, Ann Arbor, MI 48109 USA; 3 Department of Health Systems and Population Health, School of Public Health, University of Washington, Seattle, WA 98195 USA; 4 Division of Gynecologic Oncology, Department of Obstetrics and Gynecology, School of Medicine, University of Washington, Seattle, WA 98195 USA; 5 Medical Genetics, Mary Bridge Children’s, MultiCare Health System, Tacoma, WA 98405 USA; 6 Clinical Science & Innovation Department, City of Hope, Duarte, CA 91010 USA; 7 Department of Community Health Sciences, Boston University School of Public Health, Boston, MA 02218 USA

**Keywords:** Hereditary cancer, Genetic testing, Pathogenic variants, Primary care, Patient decision-making, Family communication, Cascade testing, Clinical follow-up, Population-based screening, Preventive care

## Abstract

**Background:**

Genetic testing for hereditary cancer risk can improve health outcomes for individuals and their family members. Although the use of multiplex gene panels for cancer risk is expanding in the primary care setting, little is known about patient actions following such testing. We examine participant-reported actions around clinical follow-up and discuss cascade testing with family members after a pathogenic variant (PV) is identified.

**Methods:**

Thirty-two semi-structured interviews were conducted with primary care patients who had a PV identified through the EDGE (*E*arly *D*etection of *GE*netic risk) study. Thematic analysis was used to explore participant-reported post-testing actions, followed by subgroup analysis by variant actionability.

**Results:**

Patients reported working closely with providers to decide whether to change their cancer-related care and to consider the clinical implications of their test results. Although patients’ reported promotion of cascade testing did not differ by variant actionability, those with actionable variants spoke more often of family members completing cascade testing after they discussed it. Age, sex, cost, and competing priorities influenced the perceived relevance of genetic test results and the urgency of participant-reported post-testing actions.

**Conclusions:**

In this primary care sample, the use of multiplex gene panel testing for cancer risk motivated clinical follow-up and the promotion of cascade testing.

**Supplementary Information:**

The online version contains supplementary material available at 10.1186/s12875-026-03178-9.

## Background

Cancer is the second leading cause of death in the US [1]. Genetic testing for hereditary cancer risk can inform recommendations for cancer prevention and facilitate early detection and treatment. To maximize the benefits of genetic testing, efforts are underway to expand access to preventative spaces such as primary care via multiplex gene panels. These tests allow physicians to look at a range of variants associated with common hereditary cancers without requiring them to delineate between specific single-variant tests. However, the results can be broad, and little is known about primary care patients’ actions following the receipt of results from these panels [2]. 

Hereditary cancer panels include more than 25 + genes that are differentially associated with various cancers and can identify variants with different levels of clinical actionability, which may lead to different risk management recommendations [3]. Even for the same gene, different PVs may have different associated disease risks, impacting actionability. For example, PVs in the *CHEK2* gene have different associated risks and subsequent care recommendations based on whether they are missense or truncating variants.

Ensuring the greatest utility of genetic testing relies on patients receiving and acting on evidence-based recommendations specific to their PV and clinical scenario (e.g., age, personal and family cancer history). The literature has explored clinical follow-up according to gene penetrance rather than actionability, despite many moderate genes having actionable guidelines from the National Comprehensive Cancer Network (NCCN) [4–11]. The findings according to penetrance are variable; although many studies have indicated that clinical follow-up is broadly consistent with medical guidelines, some have found evidence of overtreatment for individuals with moderate- and high-penetrance genes or variants of unknown significance [4–6, 8, 12]. How these findings translate according to variant actionability is unknown. Furthermore, the existing body of literature on patient actions following genetic testing is mostly in cancer care settings, focused on PVs associated with Lynch syndrome or hereditary breast and ovarian cancers (HBOC), with almost exclusively female participants. Our study extends these findings with evidence in the primary care setting across male and female participants and with results from a multiplex gene panel explored by variant actionability.

In addition to risk management recommendations, the promotion of cascade testing—genetic testing for the relatives of those with an identified pathogenic variant (PV)—can extend the benefits of genetic testing to family members. Although the NCCN recommends cascade testing for hereditary cancer risk, few U.S. studies offer insight into testing uptake of relatives when the proband receives their testing in the primary care versus specialty care setting [9]. 

We sought to understand what patients do after receiving PV results in the primary care setting. We specifically explored patients’ self-reported actions regarding clinical follow-up and promotion of cascade testing after receiving a PV result from a multiplex gene panel test in the primary care setting [13, 14]. We further examine participant responses by variant actionability to inform the continued use of multiplex gene panel tests for hereditary cancer risk in primary care.

## Methods

Methods and results are reported according to the Consolidated Criteria for Reporting Qualitative Research (Supplemental Materials 5) [15].

### EDGE trial design

The Early Detection of Genetic Risk (EDGE) Study was a clinical trial that tested two methods of population screening in the primary care setting: direct-to-patient (completed online) and point-of-care (in the clinic or over the phone) [13]. Participants with a significant personal or family history of cancer were offered multiplex testing at no cost. Detailed information regarding the risk assessment tool is published in a separate manuscript [13]. EDGE participants were recruited from two healthcare systems with clinics located in Montana, Wyoming, and Washington.

The panel included 29 genes associated with breast, colon, skin, ovarian, pancreatic, prostate, stomach, and uterine cancers [16]. All test results were shared through Color Health’s website, uploaded to the patient’s electronic health record (EHR), and sent to the patient’s primary care provider. Participants with a PV were offered free genetic counseling through Color Health and, depending on the health care system, were referred to outpatient clinical genetic counseling services. Relatives of participants with a PV had the opportunity to receive testing at a reduced rate. Pathogenic results were classified as actionable if they had risk-reduction recommendations in NCCN guidelines, or non-actionable if no such recommendations were provided [13]. 

Prior to receiving results, all participants with PVs were contacted by a Color Health Genetic Counselor and offered post-test genetic counseling at no cost. In some cases, a clinical genetic counselor from the health system also reached out. A report generated by Color Health was shared with both the patient and their provider via the patient’s EHR and directly through the Color Health website [13]. As such, return of results varied slightly based on who participants first engaged with. The report detailed (for both actionable and non-actionable results) that a pathogenic variant was identified, provided potential screening guidelines that may be applicable, and directed patients to speak with their provider to determine how these guidelines may vary based on their personal history. It was up to the provider’s discretion to determine what clinical recommendations to make. Providers were offered study led training on the process of identifying and following up with high-risk patients for optional continuing medical education (CME) credit (Supplemental Materials 6). Additionally, a study-designed previvor plan, which detailed information about patients’ genetic test results and included questions that patients could ask their provider, was mailed to all participants with a PV.

### Study population and recruitment

Every patient with a PV identified through the EDGE during the recruitment window from December 2021 to September 2022 (*N* = 86) was contacted to complete an initial interview intended to evaluate their feelings before receiving their results (Supplemental Materials 3). Those who completed the initial interview (*n* = 56) were contacted via e-mail or phone 6‒9 months later (2022–2023). The second interview aimed to capture participants’ self-reported actions following receipt of results, which was the focus of this analysis (Supplemental Materials 4). All invitations were sent by the interviewer (FB), who introduced herself and outlined the purpose of each study.

### Data collection

The interview guide, developed by the study team and pilot tested with co-investigators, contained questions on participants’ feelings about their genetic test results, concerns about their cancer risk, views on their overall health, behavior changes related to receiving results, discussions with family members, and communication with providers regarding results and follow-up care plans (Supplemental Materials 4). Interviews were semi-structured allowing for flexibility in conversations and reflective shifts in question phrasing and overall flow.

The interviews were conducted and recorded via Zoom by a research assistant with qualitative training and an interest in implementation work (FB), averaging approximately 43 min. Field notes were made for each interview, immediately following its completion. The interviews were transcribed, deidentified, and uploaded to ATLAS.ti for coding and analysis. Demographic data were self-reported, collected at the end of the interview, or via a survey following interview completion via UW’s HIPAA-compliant REDCap system.

### Analysis

We conducted a thematic analysis [17]. Codebook development began with deductively creating codes based on the interview guide. From there, a subset of transcripts was used to pilot deductive codes and inductively identify additional codes. The final codebook was reviewed by the study team’s qualitative research expert (ST). The transcripts were coded by two study team members (FB, HH) who met for regular reconciliation and consensus meetings, updating the codebook as needed until thematic saturation was met [18]. 

The analysis team utilized an iterative analysis approach, which began with initial data familiarization, codebook refinement, and thematic identification using codes and memos related to patient-reported changes in behavior, provider communication, follow-through with clinical follow-up recommendations, and family communication. In later stages of analysis, data were stratified by variant actionability (actionable, not actionable) to identify differences and commonalities in themes between subgroups [19]. Demographic factors were considered throughout the thematic development process. Quotes from participants with an actionable variant are denoted with an “A” (ex. A1) and those with a non-actionable variant with a “NA” (ex. NA1).

## Results

### Participant demographics

Fifty-six of the 86 participants with PVs completed an initial interview and were invited to participate in this follow-up interview. Of these, 32 completed the follow-up interview (32/56; 57% response rate). Compared with all individuals who received pathogenic or likely pathogenic results through the EDGE study, our sample was slightly younger and had a greater proportion of males. Twenty-one participants had actionable variants (65.6%), and 11 had non-actionable variants (34.4%) [20]. The interviewed participants were predominantly White (27/29, 93.1%) and female (20/32, 62.5%). The average age of the participants was 61.81 years (Table 1). Thirty-one participants (96.9%) reported they spoke with either a genetic counselor (26/32, 81.2%) and/or a provider (23/32, 71.9%).

**Table 1:** Demographic table

Characteristic		n (%) (*n* = 32)
Sex	Female	20 (62.5%)
	Male	12 (37.5%)
Gender	Woman	19 (59.4%)
	Man	10 (31.3%)
	Missing	3 (9.4%)
Age	30–45	5 (15.6%)
	46–70	18 (56.3%)
	70+	9 (28.1%)
Race/Ethnicity (*n* = 29)	White, non-Hispanic	27 (93.1%)
	Multi-racial, Hispanic	2 (6.9%)
Clinic	Billings Clinic	9 (28.1%)
	MultiCare	23 (71.9%)
Household size	Average (SD)	2.31 (0.81)
Household Income (*n* = 29)	< $75,000	10 (34.5%)
	≥ $75,000	14 (48.3%)
	Prefer not to answer	5 (17.2%)
Education (*n* = 29)	High school graduate (diploma or GED or equivalent)	3 (10.3%)
	Some college or postsecondary training	8 (27.6%)
	Bachelor’s degree (for example: BA, AB, BS)	9 (31.0%)
	Graduate or professional degree (for example: MA, MBA, JA, MD, PhD)	9 (31.0%)
Insurance* (*n* = 29)	Commercial	16 (55.2%)
	Government/military insurance	2 (6.9%)
	Public insurance (Medicaid or Medicare)	17 (58.6%)
Personal Cancer History	Yes	12 (37.5%)
	No	20 (62.5%)
Genes	Actionable (APC, ATM, BRCA1, BRCA2, CDKN2A, CHEK2**, MSH6, PALB2, PMS2, MITF)	21 (65.6%)
	Non-Actionable (CHEK2**, RAD51D***, MUTYH)	11 (34.4%)

*Multi-select questions, percentages are out of total number of respondents but may be overlapping

**CHEK2 missense mutations are classified as non-actionable while CHEK2 truncating variants are classified as actionable

***RAD51D mutations are classified as actionable in females and non-actionable in males

### Themes related to the clinical follow-up of PVs in primary care

#### Patients with actionable variants discussed and pursued changes in cancer-related care

Those with actionable variants engaged in conversations with their providers that were centered on the next steps and follow-up care. The participants thought that their genetic test results motivated their providers to refer them to specialists, “but me having the test, what I did, it was really a gotcha, because it really pushed my primary care provider (PCP) into getting that urologist. Say, ‘Okay, I know it’s probably nothing but let’s go check it out, let’s test it” (A8) and recommend cancer screening, “For the first time in my life, I’m going to say this was when I last saw my PCP several months ago, that they did do the prostate cancer screening via blood” (A1).

Ultimately, more than half of the participants reported changing their care in response to the genetic test results following conversations with their physician (Supplemental Materials 1). In the cases where genetic testing and physician follow-up led to an early cancer diagnosis, participants felt that their provider’s quick action in response to identifying a PV saved their life:

As I recall, my primary care doctor was the one who first contacted me and let me know about [genetic test results] and had a plan of further screening tests for me to do. He made a very rapid appointment in person just a couple of days later for us to discuss it… I think I would’ve a year or two from now, be[en] faced with a very horrible prognosis. And as of today… I’m in remission and hopefully will remain so for the rest of my life. - A10.

Of the participants who reported not changing their care, half had a prior history of cancer and had begun cancer surveillance prior to receiving genetic testing through the study: “Like I said, [PCP] knew I’d already had the oncologist… so nothing other than just keep following up and to have my mammograms… I had [a] mammogram, and it was discovered I had two lumps on my left breast. And so, then I’ve been through it again, had surgery and the whole nine yards” (A17). Other participants reported that they were already receiving care that was consistent with recommendations made based on their genetic test results: “The recommendations [PCP] are consistent with what we’ve already put in motion” (A15). One individual reported that older age reduced their urgency.

#### Most patients with non-actionable variants do not change their cancer-related care

Participants with non-actionable variants said that their conversations with providers and genetic counselors were centered on understanding what the results mean. Most participants with non-actionable results did not report changing their care, as exemplified in this comment: “I think the only reason why I may not [change my current care] is because they said the chances are so low… I don’t want to do unnecessary procedures… if there’s no, like the odds are not against me, I guess” (NA9; Supplemental Materials 1). When asked if there were any plans for future follow up care, another participant responded “Not at this time. I don’t have any chance [or need]” (NA11).

The participants also discussed changes to screening initiation and intervals. One participant, age 40, was considering starting colonoscopies earlier but had no definitive plan to start screening: “we [participant and provider] discussed the need to probably get a colonoscopy, based on those results, a little earlier than other people, than is recommended. So that’s all we discussed, just to be a little more diligent about monitoring and that kind of thing” (NA1). Another participant, age 67, described a situation of a non-actionable variant leading to potential overtreatment: “He [provider] said, ‘Well, you need to have your colonoscopy every five years,’ because I just changed it to 10 years because I’d been clear for 15, and so I just go back to five years” (NA2).

#### Patient age influences the relevance and urgency of clinical follow-up

Age played a direct role in participants’ reported actions related to preventative care following genetic testing. One individual indicated that their age (79 years) decreased both their urgency and their providers’ urgency for surveillance. As a result, they made no changes to their care following the identification of an actionable PV:

At my age, it’s different than if I were a really young person, and had a whole lifetime ahead of me to worry about these things… [My PCP] said if I wanted to do a home test for colon cancer, I could, and he gave me a prescription for that… He didn’t really urge me to do it… (I: Are you planning to?) I don’t think so. (I: And why is that?) Well, I guess, again, because of age. - A18.

Older participants also expressed less fear of positive results, as they had already avoided cancer for a significant amount of time. Furthermore, health concerns were more abundant in this population, so there were also competing health priorities that impacted decision-making around surveillance, as exemplified by this comment: “Plus recently, I had a heart attack, so prostate cancer is on the back of my head but not in the front. My major concern is different right now” (NA5).

Younger individuals often felt it was too early to start engaging in clinical preventative actions. When asked if they would have continued with genetic testing if it was not offered for free, one younger participant said, “Probably not. Not now. It maybe would’ve been something I would’ve done later” (NA9). However, younger individuals were uniquely concerned about the potential impact on family planning. “We talked about my potential risk was low, whether or not it could be something that my child could inherit. We talked about that as well” (NA9).

### Themes related to the promotion of cascade testing with family members

#### The promotion of cascade testing did not differ by variant actionability

Family members were often the first people participants talked to about their results. The participants’ concerns for family members’ genetic risk for cancer often outweighed their concerns about their own risk. This was in part because the probands felt that they were already doing everything they needed to do for themselves. Nearly all individuals with actionable variants discussed results with family members and encouraged them to pursue testing, e.g., “I keep reminding them and I want them to get tested” (A17). The remaining participant spoke to some family members but chose not to discuss their results with their son:

Well, I mean, my mom and one of my sisters also have tested positive for the MSH6… The one that’s the hardest conversation is with my son, because he’s 21 and we have not told him. I’m fine talking about myself, but the risk that he may have, we haven’t enforced that he should go get the testing. - A15.

Participants with actionable variants often reported that their family members ultimately completed genetic testing following these conversations. When a participant reported not speaking with family members, they indicated that the results were “just not in the front of my mind” (A2; Supplemental Materials 2).

Among those with non-actionable variants, almost all individuals discussed their results with family. Of these, a few indicated that family members completed testing following the conversation. One said, “I sent the [Color discount] link to my daughter, and she also had this genetic testing done… I strongly encouraged [my sister] to go through some testing. She might do it” (NA5).

#### Older patients perceived greater utility of actionable PV results for younger relatives

While older individuals expressed little personal concern regarding PV results, they expressed significant concern for their relatives. Just over one-third of the participants in this sample were over the age of 70, and many felt that sharing their results could benefit younger relatives. “I talked to [my] daughter six months ago or whatever…She had it done, and then since then, two of her daughters have had the test and are positive. And then I have my granddaughter that had one about three, four months ago, but she is not BRCA2” (A14).

The complex associations among age, competing health priorities, and family concerns are captured in the following quote: “I talked to my family about testing. My son is younger, and he doesn’t feel like it’s time to start thinking about it yet. My sister is older and has enough medical problems as it is” (A10).

#### Men with actionable variants had greater concern for female relatives

Broadly speaking, there was greater concern for female relatives. Men with actionable variants associated with breast or ovarian cancer were less concerned about the personal implications of their results. However, they were concerned about their female relatives. One participant explained:

I thought, honestly, with my specific results, the mentioning of female members of my family and the more significant impact it could have on them in terms of potential breast cancer, I think, was more of a concern for me than my own increased risk. -A1.

#### Cost and insurance were seen as barriers to cascade testing

While participants highlighted the benefits of genetic testing for their family members as a strong motivation for pursuing genetic testing, some worried that financial barriers may limit the potential impact. Perceptions of whether their insurance would cover the cost of genetic testing influenced family members’ decisions to pursue testing. Nevertheless, participants encouraged their family members to move forward with testing despite the cost, as in this example: “With [my] daughters, I think the daughter I mentioned who can probably get insurance coverage might go ahead with it. The other daughter, I don’t think her insurance would cover it. But I really should discuss that with her again” (A18).

## Discussion

Population-based risk assessment and testing efforts are increasingly being promoted to improve the ascertainment and management of hereditary cancer. In this study, we discuss the downstream participant-reported actions undertaken following the receipt of pathogenic variant results from population-based screening and targeted testing efforts deployed across 12 primary care clinics. This is the first study, to our knowledge, to report on participant-reported actions based on the actionability of the pathogenic result within the primary care setting. Participants who had actionable results often changed their clinical care or already had care consistent with what would be recommended (as was the case for individuals with a prior cancer diagnosis). In two cases, additional surveillance following the receipt of genetic test results led to cancer diagnoses. For those with non-actionable variants, there was minimal evidence of harm or medical actions undertaken that would not be clinically recommended, reducing concerns about potential overtreatment for these patients [8]. 

These findings enhance our understanding of the role of patient age in the follow-up actions undertaken following the receipt of genetic results. Patient age appears to influence patients’ understanding of the personal relevance of results [21–23]. The participants on the older and younger ends of the age spectrum had a reduced urgency at the time of results’ receipt; older individuals felt it was too late to engage with increased risk reduction strategies, whereas younger participants felt it was too early. However, for the benefits and utility of genetic testing to be realized, it is especially important for those identified at a younger age to take follow-up preventive action and increase surveillance. Furthermore, the necessity for provider interpretation increases when genetic testing is completed later in life. Clinical guidelines are less clear for follow-up care based on both the gene and the age of the patient. For example, for *PMS2*, continuing colonoscopies after 75 years of age is based on the provider’s discretion. While this could be partially addressed through shared decision-making, it broadly suggests that age complicates result interpretation for primary care providers and should be a targeted focus of provider education efforts, as they determine how to weigh age, competing health priorities, and actionable PVs when making clinical recommendations. For all ages, cascade testing of relatives is highly relevant [24].

Nearly all the participants had conversations with their family members about genetic testing results and promoted cascade testing. However, slight nuances in these conversations related to age, sex, and the specific PV suggest potential areas for intervention. In terms of age, the question of at what age a potentially at-risk relative should be informed arose when one participant opted not to share results with their 21-year-old son, suggesting that it was too early. A study by Bradbury and colleagues revealed that older age is strongly correlated with disclosure to offspring, although many still learn about their parents’ genetic status long before they can engage with any specific interventions [25]. A main reason cited for the delay in disclosure was waiting for the child to get older [25], but the reason behind this desire has not been thoroughly explored – is it a matter of waiting for a level of maturity, protecting a child’s right to an open future, or waiting until preventive actions are relevant? This nuance suggests a need for future research. Ultimately, work in this space can inform the development of guidelines for primary care providers around when to disclose test results with children and the key points to share to facilitate effective parent‒child cascade testing.

Both male and female participants readily promoted cascade testing with relatives, but there was greater concern for female relatives among those with HBOC-associated PVs. In addition to encouraging testing, our participants indicated that first-degree relatives then went on to complete testing. Although our study did not have access to data for relatives who completed testing, other cascade testing studies have shown that female relatives are more likely to be informed of PVs and to test [26, 27]. The gender disparity in cancer genetic testing is well established in the literature, with findings suggesting that men see less relevance in testing despite clinical implications for all genders [28, 29]. Our findings echo these results and underscore the need to communicate the relevance of genetic information to males more clearly.

The cost (both perceived and actual) of testing has been identified consistently as a barrier to genetic testing in previous studies [30]. Although some have suggested that reducing test prices may render the cost barrier less significant [25], our study revealed that many of the worries were concerns around potential costs. Genetic testing was free in our study and may have contributed to our testing rates. Moreover, to facilitate cascade testing in our study, Color Health provided all participants who had a PV with a voucher that could be distributed to first-degree relatives for discounted testing. As a result, anyone interested in pursuing testing after a conversation with the proband knew from the outset how much that endeavor would cost. According to our participant interviews, many relatives of those with actionable PVs pursued this avenue of testing. Thus, although cost reduction remains a priority, efforts to increase transparency in pricing are also critical.

The present study had several limitations that should be acknowledged. The study population was predominantly non-Hispanic White. The participants had to first complete a baseline interview and then agree to a second follow-up interview. As such, participants may reflect those who are more likely to seek and share information, which could influence family communication and the pursuit of follow-up clinical actions. The implementation of genetic risk assessment and testing in clinical practice is not uniform, so our findings must be contextualized by the resources built into our study protocol. Our interviews identified mixed evidence on the reach of study-designed previvor plans. As such, we cannot parse out the utility of the additional touchpoints or the extent to which they played a role in the follow-up actions observed for the study participants. Finally, we cannot assess the content of the provider-patient conversations, quality of recommendations made to patients or concordance with the standard of care because we do not have physician notes to review provider communication or the personal or family history context that would influence these decisions beyond genetic results.

## Conclusions

Participants with PVs from cancer genetic testing during primary care were satisfied with the information they received and believed it contributed to a more proactive approach to cancer risk reduction. Furthermore, they initiated clinical follow-up and generally promoted cascade testing in their families. The majority of participants with non-actionable variants did not change their clinical care. These data support the continued study of the implementation of cancer genetic testing in primary care populations.

## Supplementary Information

Supplementary Material 1

## Data Availability

The data supporting the conclusions of this article are included within the article and the supplemental material.

## Abbreviations

A: Participant with an actionable variant (e.g., A1)
EHR: Electronic health record
EDGE: Early detection of genetic risk
HBOC: Hereditary breast and ovarian cancers
NA: Participant with a non-actionable variant (e.g., NA1)
NCCN: National comprehensive cancer network
PCP: Primary care provider
PV: Pathogenic variant
