# Supplementary material for: Patient-reported actions following receipt of pathogenic hereditary cancer genetic test results: results from a population-based screening study in primary care
Source: BMC Prim Care. 2026 Feb 18;27:103. doi: 10.1186/s12875-026-03178-9 (PMC13020173; doi:10.1186/s12875-026-03178-9)
Supplement: Supplementary file 1 — Supplementary Material 1 [file 12875_2026_3178_MOESM1_ESM.docx]

**Supplemental Materials**

**Supplemental Material 1.** Table of clinical follow-up after receipt of genetic testing results

| **Actionable Variants (N=21)** | | | | | | **Non-Actionable Variants (N=11)** | | | | | | |
| --- | --- | --- | --- | --- | --- | --- | --- | --- | --- | --- | --- | --- |
| ID | Gene | Sex | Age | Personal Cancer hx | Representative quote | ID | Gene | Sex | Age | Personal Cancer hx | | Representative quote |
| Pursued/changed cancer prevention/management (n=14) | | | | | | Pursued/changed cancer prevention/management (n=2) | | | | | | |
| A1 | *CHEK2* | M | 41 | no | "A1: So, for the first time in my life, I'm going to say this was when I last saw my PCP several months ago, that they did do the prostate cancer screening via blood. Then she made a recommendation that I begin colonoscopies sooner, and that ideally I would've started at 40, but she wanted to have me go ahead and do one now versus waiting until I was older, given the increased risk."  Mod: have you started following up on those recommendations?  A1: Yep, I still need to get the colonoscopy scheduled, but I've already done the PSA blood screening, and will continue to do that as part of my normal health maintenance, I suppose." | NA1 | *MUTYH* | F | 40 | no | "We discussed the need to probably get a colonoscopy, based on those results, a little earlier than other people, than is recommended. So that's all we discussed, just to be a little more diligent about monitoring and that kind of thing." | |
| A2 | *CHEK2* | F | 68 | no | "So yeah, it just kind of heightened my, I guess, importance of tracking my colon. And I just had a colonoscopy within the last year or so and I'm having a mammogram next week, so."  "Like I said, she pointed out the colon cancer risk was a little... And I did receive [a colonoscopy], now that I think back based on the results, I did receive a letter suggesting that I get another and maybe more defined mammogram like every six months or something, which I didn't do, but. .. I did not discuss that part because I guess because I knew I was having a mammogram coming up anyway, so yeah." | NA2 | *MUTYH* | M | 67 | no | "He [PCP] said, "Well, here it is," so we kind of went over it. We kind of went over it. He said, "Well, you need to have your colonoscopy every five years," because I just changed it to 10 years because I'd been clear for 15, and so I just go back to five years." | |
| A3 | *CHEK2* | M | 32 | no | "And she went there and said, well, there's probably not much that you have to worry about. She probably moved my prostate exam up a few years, but that's basically it. So, it wasn't a long conversation, more, all right, well, we've made these adjustments to your long-term plan. .. I'm 33, and the recommendation for somebody that has a slightly elevated risk of prostate cancer is moving up the examinations that usually begin in your mid-40s or so up five years, I think is what I understood it to be." |  |  |  |  |  |  | |
| A4 | *CHEK2* | F | 47 | no | "...she [PCP] just sent me to the specialist to see about getting a double mastectomy and reconstruction.  Mod: Have you made any decisions about how you'll move forward with this information?  A4: I am going to do that, whenever they allow me to.  Mod: Were there any more specific recommendations made by the specialist?  A4:  No, just make sure I've got my MRIs every six months and my mammograms every six months, just rotate them." |  |  |  |  |  |  | |
| A5 | *CDKN2A* | M | 67 | no | Well, actually I've discussed them with three different doctors, now. My main provider was mostly through emails to start with, because I didn't have an appointment with him early on, and so my dermatologist knew a lot about it, about the gene anyway, and so he increased my visits from once a year to twice a year to just track and keep an eye on melanoma. My primary care provider through email had referred me to a gastro something... Gastroenterologist, I guess, and that's the appointment that I went to that was not very satisfying. He didn't seem to know much about the gene, or at least if he did, he didn't believe in the science and didn't do anything." |  |  |  |  |  |  | |
| A6 | *BRCA1* | F | 54 | no | "I actually met with a genetic counselor, and then I met with actually the OBGYN. Then they wanted me to meet with a surgeon because they actually wanted me to do a double mastectomy. I said, "Oh, I don't think I'm going to sign up for that one." But they were like, "We still want you to meet with them." They actually wanted me to meet with a plastic surgeon, and I said, "I'm not going to waste your time because I'm not going to do that." I just told them, preventatively, we're doing what... We can catch it sooner than not. I just felt like they were really on me about getting that surgery, and I was like, "I kind of think that's extreme." But I think they were trying to just make their call from the information that they had. .. but we still opted to do just the partial hysterectomies" |  |  |  |  |  |  | |
| A7 | *BRCA2* | F | 62 | no | "We discussed me seeing the OB-GYN for further testing, so I did that as well. I followed up with my OB. And in July of 2022, I had my total hysterectomy, so I had that done. I did meet with an oncology counselor, and we talked about doing the mastectomy, and we've decided to do the wait and see, watch and see route instead of just jumping right into surgery, since I'd had already had the hysterectomy done, which I think is more concerned with the BRCA gene than the breast cancer, although they're both of concern. We're testing twice a year for... the imaging, the mammogram, and then I do an MRI as well once a year, so-" |  |  |  |  |  |  | |
| A8 | *PMS2* | M | 60 | no | "But she went through all the things and that's why she started ordering all these tests with different specialists because you have to get referrals and things like that going. So, it took a little while to get into each one with the different urologist, and that's for the bladder. And then the other ones for the colonoscopy and the other things... we still got really one more testing still to go, and then one more procedure we still have to do. So, it's kind of a long road, but then I got to do certain things every six months."  "Mod: Sounds like you're doing all of the testing now, but you also have a plan in place for the future?   A8:  Right. Some are six months, some are a year. Like the bladder cancer, we have to do that testing every six months for two years, and then we go to once a year. And then the colonoscopy is every six months. And the endoscopy, I think is every six months as well. So, they kind of want to have a base and look at everything." |  |  |  |  |  |  | |
| A9 | *PMS2* | F | 68 | no | "...I just messaged him, and he [gastroenterologist] said, "Okay, because of the results, I want to check you more often than five years." So, I think I have to go back in, I want to say two years. And he wants me to do both a colonoscopy and, is it an endoscopy." |  |  |  |  |  |  | |
| A10 | *BRCA2, APC* | M | 74 | yes (after gx testing) | "He [PCP] said, "You have two oncogenes, mostly the BRCA2, and that carries a high risk for you for prostate cancer, possibly breast cancer. And the APC, which carries a small risk of colon cancer." He reviewed my PSAs. He said, "You haven't had one in a while." I said, "No, but I've had two surgeries in the meantime, which were biopsy negative." Says, "Doesn't matter. You should have PSA," And that saved my life. .. But when my PSA turned out to be 38, with a normal of 4 or 5, that's when I really got scared. That led to one thing after another, and after having been on hormone therapy now for 6 or 7 months and just completed a 28 session course of radiation therapy, my prognosis looks very good. But without having had the EDGE study participation, I would still probably have no symptoms whatsoever until I would've had stage IV disease and my prognosis would've been terrible." |  |  |  |  |  |  | |
| A11 | *PMS2* | F | 69 | yes (after gx testing) | "Because they're suggesting I have a hysterectomy as a preventative measure for uterine cancer, because I'm like 28 or 30% chance of getting that. And so I'm kind of dealing with that with my gynecologist right now. I'm going to go in this winter sometime and we're going to sit down and have a discussion about that." |  |  |  |  |  |  | |
| A12 | *MITF* | F | 51 | yes | "Right now I'm still getting scanned for colon cancer follow up, and then after that is over, when they've said that we are not doing that anymore, then he will order probably annual ultrasounds of my kidneys to make sure that there isn't cancer growing there. .. Well, the other part of it was the skin cancer, and I'm already pretty on top of that, and he [PCP] knows that." |  |  |  |  |  |  | |
| A13 | *PALB2* | F | 54 | yes | "There's another association with the PALB2 is a chance at pancreatic cancer, and so, he's very good at having me do kind of some screening for that just to catch... It's not a very big percentage that I would get it, but if for some reason I did, he would want to catch it obviously, very early. So because of that, I don't think... I haven't been in touch with anyone about breast cancer stuff at all since I had the double mastectomy. So I'm assuming that's kind of a done deal, but the pancreatic is something that they just want to make sure that is okay." |  |  |  |  |  |  | |
| A14 | *BRCA2* | F | 80 | yes | "Well, my main doctor, we mainly talked about the mammograms, the breast cancer. I thought about having bilateral mastectomy, but [back] then decided I don't want to do that right now. I also talked to my surgeon. Well, I'd gotten the mammogram, and they had found a nodule in there about a year ago. I talked to him and actually, the surgeon said he really didn't think that I had BRCA2. I hadn't got this results back yet. I don't why he thought that. He said, looking at what he had seen, there was nothing to worry about. Don't do anything for a year. That's what he told me. Two months later, he called me back and said he did want me to get the bilateral [mastectomy], so I did get that then. Since then, everything is okay, so I'm still just, whatever comes." |  |  |  |  |  |  | |
| Continued existing cancer prevention/management (n=6) | | | | | | Continued existing cancer prevention/management (n=6) | | | | | | |
| A15 | *MSH6* | M | 51 | no | "The recommendations she had are consistent with what we've already put in motion, which is making sure... The biggest one is that I'm working with a gastroenterologist and doing an annual colonoscopy because of the result, and then an endoscopy every three years." | NA3 | *MUTYH* | F | 69 | yes | "I ran that question by her first, and she said, "No, because the Cologuard is usually every year, but the colonoscopy is once every 10 years." So, she said, "In regards to your case, being in a minority of, not at high risk, then no, you should be fine with Cologuard." So, then I took that information, had a discussion with my provider, and she agreed, so that's how we went ahead with the Cologuard." | |
| A16 | *APC* | M | 63 | no | "Discussed? The results and pretty much said that we're on the right course on keeping track of the polyps through the sigmoidoscopy and endoscopy... And then another appointment, an in-person appointment a few months with her, and the upper endoscopy, which just I'm due on the five-year cycle." | NA4 | *MUTYH* | M | 71 | no | "NA4: ...he [PCP] said the good thing is that my risk wasn't that elevated…  Mod: Were there any recommendations for follow up care?  NA4: Just my normal. He had said go and do my normal colonoscopy, have that done on time." | |
| A17 | *ATM* | F | 81 | yes | "Like I said, she [PCP] knew I'd already had the oncologist... so nothing other than just keep following up and to have my mammograms... I had mammogram and it was discovered I had two lumps on my left breast. And so then I've been through it again, had surgery and the whole nine yards." | NA5 | *CHEK2 missense* | M | 69 | yes | "I talked to my urologist about this and my primary care provider, so we both agreed that, yeah, it is a mutation. But he mentioned something that I need to do this colon cancer screening more often but then we decided, no, I'll just keep the regular schedule." | |
| A20 | *BRCA1* | F | 45 | yes | "Mod:  So you had done genetic testing prior to your participation in the EDGE?  A20:  Yes. When I was 38, I had breast cancer.  Mod: How did you receive your [EDGE] results?  A20: I received them by mail... And then I did review them with my oncologist...  I asked about the other variable [PMS2 VUS], what that means, and I think it says it's inconclusive in there or however it's worded, so she just said it's nothing to worry about." | NA6 | *MUTYH* | F | 71 | yes | "I want to sit down with that primary and see what they're going to do on top of the tests I already take. I already take a yearly mammogram. I already take a yearly MRI. And so I want to get all of those tests coordinated so that I'm not doing a lot of them. They're all in an order. For example, I'm supposed to do an MRI every six months and then six months later I do a breast thing." | |
| A22 | *BRCA2* | F | 53 | yes | "Mod: Were there any specific recommendations made by your provider?  A22: No, not really, because I've already had a total hysterectomy... we've already pretty much taken any steps that he would've recommended to totally eradicate that risk.  Mod:  You mentioned though that there were some screenings and you've already done those screenings. What screenings were those?  A22: He wanted me to go in and have a mammogram, which I already did, and it came back a hundred percent A-okay. " | NA7 | *MUTYH* | F | 76 | no | "NA7: I did talk with him [PCP]. He just said, "Go to your next colonoscopy." That was his remark, and I agreed.  Mod:  Did he change the frequency of getting those colonoscopies or is just keeping you on the same schedule you were already on?  NA7:  I think it's appropriate to be on the same schedule that I'm on. " | |
| A21 | *CHEK2* | F | 66 | yes | "Mod: Do you plan on discussing the results of that test at your next appointment?   A21:  Not really. I mean, if she brings it up I will, but I don't have any breast tissue left, so it's not like I think about it that much. .. I mean, I think it will probably come up 'cause we'll talk about when I should have my next colonoscopy and that's, the CHEK2 has to do with that more. So, it could be that we talk about it that way, but that would be the only thing, probably." | NA8 | *MUTYH* | F | 67 | no | "Mod: Do you have a plan in place for follow-up care?  NA8:  Yeah. Like I said, the colonoscopy. Yeah. And then, however often they recommend after that.  Mod:   Have you had discussions about how often they'll want you to be getting it moving forward?  NA8:  No, but before it was every five years. I don't see it changing unless I have something when they do it." | |
| Did not prompt clinical follow-up (n=1) | | | | | | Did not prompt clinical follow-up (n=3) | | | | | | |
| A18 | PMS2 | F | 79 | no | "At my age, it's different than if I were a really young person, and had a whole lifetime ahead of me to worry about these things… [my PCP] said if I wanted to do a home test for colon cancer, I could, and he gave me a prescription for that. Oh, I can't remember what it's called. But you send away for the materials, and yeah. So the doctor didn't express any urgency on me doing any... He didn't really urge me to do it is, I guess, what I'm saying.  Mod:  Are you planning to?  A18: I don't think so.  Mod:  And why is that?  A18:  Well, I guess, again, because of age." | NA9 | *MUTYH* | F | 31 | no | "NA9: I think it got better when I chatted with one of the providers [from Color], and she said the chances were low for it, and so that kind of made it go, ‘Okay, well, I don't really need to worry about it as much’  Mod: Do you plan on meeting with your PCP?  NA9: I think the only reason why I may not is because they said the chances are so low. That might be... I don't want to do unnecessary procedures and unnecessary if there's no, like the odds are not against me, I guess." | |
|  |  |  |  |  |  | NA10 | *MUTYH* | F | 78 | no | "NA10: I didn't see anything to me that seemed that alarming.  Mod: do you plan to discuss the results at that appointment?  NA10:  Well, I could ask her if she got them, but I don't know. I didn't print them out, so I don't know how I would show her if she didn't receive them.  Mod: What are any steps you plan to take because of this genetic test result?  NA10: Well, I've tried to have a healthier outlook and a healthier diet. I guess that would be part of it." | |
|  |  |  |  |  |  | NA11 | RAD51D | M | 74 | no | "I haven't had the chance or the need, or the anxiety to pursue it any more than just what the people in Color have made some recommendations and there were some forms and stuff. So beyond that, I think everything is just riding along. Again, the results were neither surprising or not."  "Mod: And then in terms of follow up care, future follow-up care, are there any increased screenings or additional appointments that you plan to get?   A19:  Not at this time. I don't have any chance. I continue to think about it and do a little bit of research about it... So no, I have no real follow up except to be open-minded and receptive to the next news." | |

**Supplemental Material 2.** Table of promotion of cascade testing after receipt of genetic testing results

| **Actionable Variants (N=22)** | | | | | | | **Non-actionable variants (N=10)** | | | | | | |
| --- | --- | --- | --- | --- | --- | --- | --- | --- | --- | --- | --- | --- | --- |
| ID | Gene | Sex | Age | Personal Cancer hx | | Representative quote | ID | Gene | Sex | Age | Personal Cancer hx | | Representative quote |
| Yes, and family members pursued testing (n=9) | | | | | | | Yes, and family members pursued testing (n=2) | | | | | | |
| A17 | *ATM* | F | 81 | yes | "I was just anxious for, especially, the closer family members to have the testing done. Now, my daughter, I only have one daughter, and she had the test and hers was negative, so that was good… And I have two sons, but they have yet to be, I keep reminding them, you really need to go and be tested because they both have daughters so far. So I told both of the daughters, my granddaughters, I said, ‘you might want to get tested because your dads probably aren't.’" | | NA5 | *CHEK2 missense* | M | 69 | yes | "As I mentioned, I sent the link to my daughter and she also had this genetic testing done... I strongly encouraged [my sister] to go through some testing. She might do it. I also sent her a link but she did not use it, but she went back to Russia and probably she'll be doing some genetic testing there but yeah, so that's all I know." | |
| A20 | *BRCA1* | F | 45 | yes | "My sister and my son have done genetic testing." | | NA2 | *MUTYH* | M | 67 | no | "My son's ignoring it. Both of my brothers have ignored it until recently, and the one that has issues is still, he's dealing with his own issues, so he's not really interested in getting the study, but I did just recently get my younger brother to do the genetic testing to you guys. He's proceeded with doing that. I don't think he's got his results back yet. Then of course, my oldest daughter works at [Healthcare System], so she's already taken care of that and found out she doesn't carry the gene, but my other two daughters both do. We've had discussions about that. Then the one daughters found another gene that's got genetic deformity that they had tested for, but they didn't test me for it, so I don't know if I've got it or not." | |
| A14 | *BRCA2* | F | 80 | yes | "Mod:  Who did you talk to about your positive result?  A14: Well, my daughter yesterday, because she does have BRCA2 and she's got two daughters that have it. I talked to her and we all have the same variant. My daughter, her two daughters, and my sister and me all have it, so we were talking about that yesterday.  Mod: Did you talk to any of them about getting tested? It sounds like many of them already have been tested and have results.  A14:  I talked to the daughter six months ago or whatever, I've talked to her. She had it done, and then since then, two of her daughters have had the test and are positive. And then I have my granddaughter that had one about three, four months ago, but she is not BRCA2." | |  |  |  |  |  |  | |
| A21 | *CHEK2* | F | 66 | yes | "A21:  But my six sisters, we've all talked about it.  Mod:  You mentioned that all of them did go through with the $50 testing through Color, correct?  A21:  Yeah. It was funny how it happened, 'cause when I offered it, I said, "Just reply privately to me and we'll make it happen." I didn't want anybody to feel pressured. So then, we have a weekly family call on Zoom, and so then as people got their results in, they would start blabbing about it. And then the ones that didn't were like, ‘What's that again?’ And they couldn't... 'cause it was just one email. And "Well, maybe I'll do that." And so, they kind of all did it in dribs and drabs, and then the last one did it fairly recently." | |  |  |  |  |  |  | |
| A12 | *MITF* | F | 51 | yes | "Mod: Have you discussed your results with any of your family members?  A12: Oh yeah. I had them all tested… Color offers a $50 testing to any family member over 18. I had both my sisters and my parents." | |  |  |  |  |  |  | |
| A13 | *PALB2* | F | 54 | yes | "My sister was also tested, and she also has the PALB2 mutation, so yeah. And she has the same doctor as me, too... I do have another sister who I'm not in contact with very much. And so, I believe she had the test, and I believe she was positive as well for that." | |  |  |  |  |  |  | |
| A5 | *CDKN2A* | M | 67 | no | "Mod:  And who have you talked to about your positive result?   A5:  My wife and both children... both of the kids got tested and yeah, just worth concern probably but... Oh, I did talk to my sister too. I forgot about that. She actually got tested too, I think." | |  |  |  |  |  |  | |
| A8 | *PMS2* | M | 60 | no | "My sister took it real serious because she did the testing. My son, probably not at first, but we did talk him into doing the testing. So that was good. And my [other]sister, I think she's more at risk than I am, but I don't think she's done anything about it." | |  |  |  |  |  |  | |
| A9 | *PMS2* | F | 68 | no | "...my children have now had genetic tests done. Well, actually my daughter. She came back positive for the PMS2 gene. And my son, who has had cancer, well, he had a polyp that was found to be cancerous, and they've now, because of my results, have referred him to a genetic counselor."  "My mother, my sisters, my children... Well, we went over the results and that I was positive for the PMS2. And I gave them, actually, a copy of the results, and then they took it to their doctor. And then their doctors decided whether they needed additional testing or genetic counseling, in the case of my son." | |  |  |  |  |  |  | |
| Yes, discussed with family but no known follow-up (n=11) | | | | | | | Yes, discussed with family but no known follow-up (n=8) | | | | | | |
| A11 | *PMS2* | F | 69 | yes (after gx testing) | "... And [geneticist’s name] actually sent her an invite for a genetic test for her and her family. She said, "No, she wasn't interested... her biggest thing is the COPD because she's on oxygen all the time. So that's a huge thing for her. And I think she doesn't want the stress, which I don't blame her. And I didn't push it." | | NA4 | *MUTYH* | M | 71 | no | "When I discuss it with my daughter, she was blasé, because she's a nurse, so therefore she's an expert at everything." | |
| A10 | *BRCA2, APC* | M | 74 | yes (after gx testing) | "Mod:  Who did you talk to about the positive result?  A10:  My sister and my son and anybody else who had listen, but they're not family members.  Mod: How did they respond?  A10:  My sister responded in a sympathetic and caring way, but felt that with all of her medical problems ongoing, that it would have little impact in changing what she's already much involved with. And my son deferred thinking about it, I think for now, feeling he had more important decisions and goals in the near future." | | NA1 | *MUTYH* | F | 40 | no | "After I got the test results, I let my sisters and my parents know, and then I let a lot of my cousins know... And so it was just more of, ‘Oh, okay, this is an interesting thing. I should pass this on so that people are aware’...I did tell my sisters if they wanted to get the test too, that I think that that was available to them. And then I sent them the link." | |
| A18 | *PMS2* | F | 79 | no | "My daughters were more interested. One daughter, her health insurance will cover genetic testing. So she's thinking about doing that... Well, with my brothers, they probably don't see a real need for it. With the daughters, I think the daughter I mentioned who can probably get insurance coverage might go ahead with it. The other daughter, I don't think her insurance would cover it. But I really should discuss that with her again." | | NA6 | *MUTYH* | F | 71 | yes | "Mod: have you discussed your results with any of your family members?  NA6: Yes, I have. With kids and with siblings.  Mod: Okay. And how did they respond?  NA6: They're all going to do something about it, but nobody's done anything yet.  Mod: Did you talk to them about getting tested or did they express any interest?  NA6: I did and they said they would. And I sent them all the information, but I don't think anyone's done anything." | |
| A1 | *CHEK2* | M | 41 | no | "My parents, and then of course for the sent out notification to the females in my immediate family about their potential for increased risk as well... So, I passed along information and I think Color includes some good information to send to them. I actually don't know if they followed up on it, but they did confirm receipt and that they appreciated it and were going to follow up, I just don't know if they have." | | NA7 | *MUTYH* | F | 76 | no | "Well, I think my sister couldn't remember. She apparently was tested, but I don't know if she was positive. I couldn't get that from her. And my husband just kind of rolled with it. I don't have cancer. I just have a little bit easier time of having colon cancer. Well, it's certainly true about a lot of things in life, so... I just talked to my niece and I think she had so many things that she was doing that it wasn't anything she was interested at this point. " | |
| A16 | *APC* | M | 63 | no | "A16: It was good to share my thoughts with my sister. Hopefully, it comforted her.  Mod: And what exactly did you guys talk about?  A16: Results… Getting tested. And for my son… just ya know probably… get tested and probably get his own genetic, maybe... Possibly could escape the cycle of yearly testing that I've been doing for so long.  Mod: So, you did talk to your son about possibly getting the genetic testing as well?  A16: Yes." | | NA10 | *MUTYH* | F | 78 | no | "Mod: Have you discussed your results with any of your family members?  NA10:  Yes, my sister.  Mod: And how did they respond?  NA10: They shrugged their shoulders like, 'So.'" | |
| A3 | *CHEK2* | M | 32 | no | "I suppose my results were that I was slightly elevated myself, but that the women in my family were more elevated due to the certain types of cancers that showed up in my genetic material. So I think the first thought I had was concern for my sister, I guess... I said, yeah, you should maybe look into getting this sort of test yourself to my sister, knowing that the mutation is not guaranteed to be in her genes as it is in mine. So I can't say for sure what their follow-up was, but I let them know, this is an interesting thing that I found out that has ramifications for you." | | NA3 | *MUTYH* | F | 69 | yes | "Mod: Did you talk to them about getting tested or did they express any interest?  NA3:  Oh, my sister's 80, so she doesn't need that. That niece is a nurse, so she kind of knows her medicine stuff, I don't need to tell her what to do. But I just told them. Oh yeah, I told friends. I told friends that if they got offered a free test like this, to make sure they took it, that's for sure." | |
| A4 | *CHEK2* | F | 47 | no | "Mod: Was there anybody that you talked to about your positive result who had not already been tested  A4: My daughter. I have a daughter who's 30.  Mod: Did she want to get tested?  A4: She does, but she was waiting because you have to make sure you have all your life insurance and all that before your testing, so I told her to make sure she has all of that before she does her testing." | | NA8 | *MUTYH* | F | 67 | no | "My sister acted like she already had a test and knew, but I think she just acted like that. She doesn't like me being the first in anything. And then my brother was like, ‘Oh, I'm too old. I haven't had problem yet,’ because he's like... How old is he? 73 or 74. And my son was like, ‘No.’ It was just recently. I told him on his 46th birthday. I asked him. I didn't tell him. I asked him if he's had a colonoscopy. He like, ‘No.’ And I said, ‘No, you need to get one.’” | |
| A6 | *BRCA1* | F | 54 | no | "Mod: Did you talk to them about getting tested or did they express any interest?  A16: Oh, I talked to them. Told them, "Hey, this is what you need to do." Made it their choice if they wanted to grade. But because my family circle is so small, there wasn't really very many that I had to talk to.  Mod: Did most of them want to get testing?  A16: Well, my daughter already had. My son was going to do the stuff that he needed to do. My sister was not. Then her daughter, I think, was going to, so I was going to send her my information so that she had all that." | | NA11 | *RAD51D* | M | 74 | no | "I have had some interesting conversations with my brother and two sisters... ends up that both them and their children, and in one case, their children's children knew of course more about it than we did and had done their research. One of their children then was trying to have babies and had done an awful lot of research, including their own genetic testing. So I don't think there were any surprises. And I think that one of the conversations ended up well with a niece saying, ‘well, we knew that’, or ‘we had already found something else’ and et cetera."  "Mod: Did they go forward with testing themselves?  A19: Yes, we talked quite a bit about it. And again, I might have even read a sentence or two from the communication that I've gotten. We talked about the $25 fee for them to participate. None of them wanted to." | |
| A7 | *BRCA2* | F | 62 | no | "Mod: Who have you talked to about your positive result?  A7: My parents, my children, my husband, my nieces, my nephew, briefly. I need to follow up with him some more. He has a daughter, well, boys too, I guess. He has a son and a daughter. So yeah, it's important to make them aware of it as well.  Mod: And did you talk to them about getting tested, or did they express any interest?  A7: Yeah, I did talk to them about it, and that's one of the things that I need to sit down and add their information to the Color system and get them sets of testing kits as well, if I can still do that." | |  |  |  |  |  |  | |
| A22 | *BRCA2* | F | 53 | yes | "Mod: Who have you talked to about your positive results?  A22: All of my seven siblings.  Mod: And how did they respond?  A22: They were like, "Okay, cool. Yay, our stuff all matches," except for my one sister who has a higher breast cancer risk than all of us for some reason." | |  |  |  |  |  |  | |
| A15 | *MSH6* | M | 51 | no | "Well, I mean, my mom and one of my sisters also have tested positive for the MSH6, so we're talking more from a supportive... Everybody's supportive, I guess, that would be the better way to say it... The one that's the hardest conversation is with my son because he's 21 and we have not told him. I'm fine talking about myself, but the risk that he may have, we haven't enforced that he should go get the testing. We want him to be able to make that decision." | |  |  |  |  |  |  | |
| Did not discuss with family (n=1) | | | | | | | Did not discuss with family (n=1) | | | | | | |
| A2 | *CHEK2* | F | 68 | no | "Mod: Have you discussed your results with any of your family members?  A2: No.  Mod: Okay. Is there any particular reason why you're hesitant to share this information with them?  A2: No, no reason, it's just not in the front of my mind... But it's a good suggestion because my other sister is a breast cancer survivor and I think she'd be very interested, so, good idea." | | NA9 | *MUTYH* | F | 31 | no | "Mod: Have you discussed your results with any of your family members?  NA9:  My husband.  Mod:  Anyone else?  NA9:  No.  Mod: What did you talk about?  NA9:  We talked about my potential risk was low, whether or not it could be something that my kid could inherit. We talked about that as well.  Mod:  And did you talk to them about getting tested at all?  NA9:  I did. And we're still undecided about when will be a good time for that, mostly because of cost." | |

**Supplemental Material 3**. Baseline Interview Guide

**EDGE Project BASELINE PREVIVOR INTERVIEW GUIDE**

# Introduction

Thank you for participating in the EDGE Project- **E**arly **D**etection of **GE**netic risk. This interview is a follow-up to the genetic testing that you recently completed. The EDGE project is testing a comprehensive hereditary cancer assesment program, using a population-level approach in the primary care setting. As you know, the program is collecting personal and familial cancer history, assessing an individual’s risk of “hereditary” cancers (that can sometimes be passed through family-genes), and providing genetic testing to those found at risk.

As a patient who has been determined to have a family risk of hereditary cancer, we would like to get your perspective on the participation process. Your comments and observations will be combined with those from other patients, and your contributions will remain confidential.

I am going to start recording the audio so that we have a transcript of our conversation.

[Turn on recorder]

This is (interviewer name) and I am speaking with (patient name) for a baseline interview.

Sound ok? Let’s begin.

**What motivated you to participate in this program?**

- Were you motivated by:
  - - - Curiosity? Desire to be informed?
      - Feelings of obligation to biological relatives? Can you describe these?
      - Do you know anyone with cancer? Who? How close are you?
      - Did you experience any pressure to do genetic testing from anyone: your PCP? Friends? Family members?

# Primary Care Provider Assessment

**We have in our records _______ as the name of your primary care provider. By Primary care provider we mean the provider that you go to with general health and medical issues. Is that what you think? If no, who is your primary care provider?**

OK, the next questions ask about your relationship with your primary care provider.

## Quantitative questions (SGCS)

**Instructions**: In an effort to continuously monitor and improve the quality of **genetic services** delivered to patients at this health care organization, I would like to ask six questions regarding the appointment you recently completed with your PCP/Genetic Professional. I will read each statement very carefully. Please tell me how much you agree or disagree with each statement by choosing the response that best describes your feelings, from: Strongly disagree, Disagree somewhat, Uncertain, Agree somewhat, and Strongly agree. Your honest responses will be kept strictly confidential, and they will not be shared with your provider. Thank you for assisting us.

|  | **Strongly disagree** | **Disagree somewhat** | **Uncertain** | **Agree somewhat** | **Agree strongly** |
| --- | --- | --- | --- | --- | --- |
|  |  | | | | |
| 1. My PCP seems to understand the stress I was facing. | 1 | 2 | 3 | 4 | 5 |
| 2. My PCP helps me to identify what I needed to know to make decisions about what would happen to me. | 1 | 2 | 3 | 4 | 5 |
| 3. I feel better about my health after meeting with my PCP. | 1 | 2 | 3 | 4 | 5 |
| 4. In general, time with my PCP is about the right length of time I need. | 1 | 2 | 3 | 4 | 5 |
| 5. My PCP is truly concerned about my well-being. | 1 | 2 | 3 | 4 | 5 |
| 6. Sessions with my PCP are valuable to me. | 1 | 2 | 3 | 4 | 5 |

**That is the end of that section. Thank you for your responses. Now we will continue to the remaining interview questions.**

**Can you tell me about your relationship with your PCP?**

Probe for:

- - Do you see the same provider for your general health maintenance & health issues?
    - How long have you seen this provider?
  - How confident are you in your PCP’s ability to guide preventive health interventions?
  - Are you able to schedule an appointment with your PCP when needed?
  - (Perception of PCP’s genetic literacy) How knowledgeable is your PCP when it comes to medical genetics? And how confident are you that they have the knowledge to deal with your issues?

Now we go on to more general questions on Health behavior changes:

**You provided information about your personal and family history earlier in this program. Since then, have you made any changes to medication, health behavior, or lifestyle?**

*Probe for changes in dietary, physical activity, medication/vitamin regimen, sleep habits, and stress reduction

- Have you had any other tests or check-ups as a result of being told this information? [Probe for intentions of further screening tests, such as mammogram, pap smear, colonoscopy?]

**General questions of current feelings/status:**

**In general, would you say your health is:**

- **Excellent**
- **Very Good**
- **Good**
- **Fair**
- **Poor**

**Next, I am going to read you a series of statements regarding your feelings over the past 7 days with respect to your experience with cancer risk. After I read each statement please tell me if you have thought similarly in the past week on a scale of 1 to 4. 1 being not at all, 2 being rarely, 3 being sometimes, or 4 being often. Do you have any questions before we begin?**

|  | **Not at All** | **Rarely** | **Sometimes** | **Often** |
| --- | --- | --- | --- | --- |
|  |  | | | |
| 1. I thought about my cancer risk when I didn’t mean to. | 1 | 2 | 3 | 4 |
| 2 I avoided letting myself get upset when I thought about my cancer risk or was reminded of it. | 1 | 2 | 3 | 4 |
| 3. I tried to remove my cancer risk from memory. | 1 | 2 | 3 | 4 |
| 4. I had trouble falling asleep or staying asleep because of pictures or thoughts about cancer that came into my mind. | 1 | 2 | 3 | 4 |
| 5. I had waves of strong feelings about my cancer risk | 1 | 2 | 3 | 4 |
| 6. I had dreams about cancer risk. | 1 | 2 | 3 | 4 |
|  |  | | | |
| 7. I stayed away from reminders of my cancer risk. | 1 | 2 | 3 | 4 |
| 8. I felt as if it it hadn’t happened or wasn’t real. | 1 | 2 | 3 | 4 |
| 9. I tried not to talk about my cancer risk | 1 | 2 | 3 | 4 |
| 10. Pictures about cancer popped into my mind. | 1 | 2 | 3 | 4 |
| 11. Other things kept making me think about my cancer risk. | 1 | 2 | 3 | 4 |
| 12. I was aware I still had a lot of feelings about it, but I didn’t deal with them. | 1 | 2 | 3 | 4 |
|  |  | | | |
| 13. I tried not to think about my cancer risk. | 1 | 2 | 3 | 4 |
| 14. Any reminder brought back feelings about my cancer risk. | 1 | 2 | 3 | 4 |
| 15. My feelings about my cancer risk were kind of numb. | 1 | 2 | 3 | 4 |

**That is the end of that section. Thank you for your responses. Next, we will go over the**

**final set of interview questions.**

**What was it like when you were informed that you were eligible for genetic testing?**

Did you seek any further information about your genetic screening on your own?

- - - What methods did you use to gather more information? (internet, friends/family, health professionals?)
  - How would you rate your access to resources that provide reliable information?
  - Since you were informed about your risk, have you been concerned about anything in particular? (i.e. finances, potential for future illness, biological relatives’ health, etc…)
  - How do you feel about participating in this program?

Finally, we can talk about communication and attitudes about evaluating for genetic risk for cancer:

**Who have you told about your possible genetic risk for cancer?**

- Did you talk to them about getting tested? Do they want to get tested?
- Do you think genetic screening for cancer should be a routine primary care practice?
  - Any concerns? (Financial, anxiety, ease of getting life or long term care insurance, etc.?)
- Who should be responsible for telling patients about genetic testing for cancer risk?

**What else would you like to share about your experience with cancer genetic testing?**

**Have you received your results for the cancer genetic testing?**

- Were you able to access your portal to view them?

**Thank you so much for taking the time to speak with me.**

- *Describe the process for receiving and redeeming the gift card.*
- *Ask the patient to inform study staff if they have a change in contact information*
- *They will be contacted to schedule a short-term follow up interview in a few months*

**END OF INTERVIEW.**

**Supplemental Material 4**. 6-9 Month Follow-Up Interview Guide

**EDGE 6 Month Follow-Up Previvor Interview Guide**

# Introduction

Thank you for participating in the EDGE Project- **E**arly **D**etection of **GE**netic risk. This interview is a follow-up to the genetic testing that you completed approximately 6 months ago. To refresh your memory, The EDGE project is testing a comprehensive hereditary cancer assesment program in the primary care setting. As you know, the program is collecting personal and family cancer history, and providing genetic testing to individuals with a personal or family history of cancer.

As a patient who has a cancer risk gene, we would like to learn more about your experiences after you received your test result. Your comments and observations will be combined with those from other patients, and your contributions will remain confidential.

Please note, the questions in this interview are standardized and are not specific to your individual test result. They may or may not be relevant to your personal experiences following genetic testing.

I am going to start recording the audio so that we have a transcript of our conversation.

[Turn on recorder]

This is (interviewer name) and I am speaking with (patient name) for a follow-up interview.

Sound ok? Let’s begin.

# Initial Reaction

**How did you feel when you received your test result?**

Prompt:

- What emotions did you experience?
- What were you expecting (and why)?
- How, if at all, did your feelings change over time?

**How did you receive your results?**

Prompt:

- Color Portal?
- Patient portal?
- At your appointment with your PCP?
- Over the phone from your PCP?
- Genetic counseling appointment with Color?
- Genetic counseling appointment with the clinic?

# Primary Care Provider Assessment

The next questions ask about your relationship with your primary care provider

**Have you had the opportunity to discuss your results with your primary care provider?** *(prompts continued on next page)*

Prompts:

**If yes,**

1. What was discussed with your provider?
   1. Were there any specific recommendations made by your provider?
      1. If so, have you started following up on any of those recommendations? ￼
   2. Do you have a plan in place for follow up care?
      1. Have you made any decisions about how you will move forward with this information?
2. Did you receive a referral to a specialist or genetic professional outside of the Color genetic counselor?
   1. Were there any specific recommendations made by the specialist/genetic professional?
      1. If so, have you started following up on any of those recommendations? ￼
   2. Do you have a plan in place for follow up care?
3. Do you mind sharing if there were any additional costs for the follow-up care you have received (or plan to get)?

**If no,**

1. Do you plan on meeting with your PCP?
   1. Is there a specific reason you would not follow-up about your results with your PCP
      1. Too busy
      2. Not ready to have that conversation
      3. Did you go straight to a specialist?
2. What are any steps you plan to take because of this genetic test result?
3. What are concerns you have about any follow up care you plan to get?
   1. Do you have any cost concerns for the follow-up care you plan to get?

**How confident are you in your PCP’s ability to guide preventive health interventions?**

- (Perception of PCP’s Genetic Literacy) How knowledgeable is your PCP when it comes to medical genetics?
- And how confident are you that they have the knowledge to manage your health issues?

**You may have mentioned this earlier, but did you meet with a genetic counselor?**

- If yes,
  - Did you meet with a counselor at Color Health?
    - If yes,
      - Can you tell me a little bit about that appointment? ￼
      - What kind of information did you review?
      - Was the counselor able to answer all of your questions?
        - How satisfied were you with their response?
      - Looking back, is there anything that you wished you discussed with the genetic counselor during your visit?
  - Did you meet with a genetic counselor from your healthcare system?
    - If yes,
      - Can you tell me a little bit about that appointment? ￼
      - What kind of information did you review?
      - Was the counselor able to answer all of your questions?
        - How satisfied were you with their response?
      - Looking back, is there anything that you wished you discussed with the genetic counselor during your visit?
    - If no,
      - Why is that?
      - Did you try to set up an appointment and it failed? (Do you recall Color reaching out to try and schedule an appointment?)
      - Do you plan to meet with a counselor in the future?
        - If no, are there reasons why you don’t want to meet with a counselor?

**For a patient who HAS discussed their results with their provider:**

In an effort to monitor and improve the quality of genetic services at this health care organization, I would like to ask six questions regarding the most recent appointment you have had with your PCP – would that be the meeting you had to discuss your results?

**For a patient who has NOT discussed their results with their provider:**

In an effort to monitor and improve the quality of genetic services at this health care organization, I would like to ask six questions regarding the most recent appointment you have had with your PCP.

**Instructions:**

I will read each statement very carefully. Please tell me how much you agree or disagree with each statement by choosing the response that best describes your feelings, from: Strongly disagree, Disagree somewhat, Uncertain, Agree somewhat, and Strongly agree. Your honest responses will be kept strictly confidential, and they will not be shared with your provider. Thank you for assisting us.

|  | Strongly disagree | Disagree somewhat | Uncertain | Agree somewhat | Agree strongly |
| --- | --- | --- | --- | --- | --- |
|  |  | | | | |
| 1. My PCP seems to understand the stress I was facing. | 1 | 2 | 3 | 4 | 5 |
| 2. My PCP helps me to identify what I needed to know to make decisions about what would happen to me. | 1 | 2 | 3 | 4 | 5 |
| 3. I feel better about my health after meeting with my PCP. | 1 | 2 | 3 | 4 | 5 |
| 4. In general, time with my PCP is about the right length of time I need. | 1 | 2 | 3 | 4 | 5 |
| 5. My PCP is truly concerned about my well-being. | 1 | 2 | 3 | 4 | 5 |
| 6. Sessions with my PCP are valuable to me. | 1 | 2 | 3 | 4 | 5 |

That is the end of that section. Thank you for your responses. Now we will continue to the remaining interview questions.

# Insurance Coverage and Cost Related to Genetic Testing

Now we are going to ask you some questions regarding the cost of genetic testing, your insurance coverage, and related follow-up care.

**How do you feel about the cost of genetic testing?**

1. If the test was not offered for free, would you have been concerned about the cost?
2. Would you have continued with genetic testing if it was not offered for free?
   1. Why or why not?
3. What is the maximum price you would pay out-of-pocket for this genetic test, if it had not been offered for free?

Moving on from the cost of testing, the following questions are related to insurance coverage.

**Do you have insurance?**

If yes,

1. What type of insurance do you have?
   - 1. Are you happy with your current insurance policy? Do you feel that they adequately cover the cost of the type of medical care you want to receive?
2. Do you think your insurance would have covered the cost of genetic testing?
3. Were you responsible for any copays for your follow up care?
4. After receiving a positive genetic test result, was your insurance more willing to cover the cost of follow-up care?

If No,

1. Did your lack of coverage impact your decision to proceed with follow-up care?
   1. Why or why not?
2. Do you think your insurance would have covered the cost of genetic testing?

# Behavior and Mental Health Assessment

**In general, would you say your health is:**

- Excellent
- Very Good
- Good
- Fair
- Poor

**Since receiving your genetic test result, have you made any changes to medication, health behavior, or lifestyle?**

Prompts:

- Probe for changes in dietary, physical activity, medication/vitamin regimen, sleep habits, and stress reduction

If no explanation is given as part of the response:

- Probe with:
  - Can you elaborate more on why you view your health as [patient’s response]?
  - Have any other health related events occurred since receiving your results?
  - How has this result affected how you view your overall health?

**Next, I am going to read you a series of statements regarding your feelings over the past 7 days with respect to your experience with cancer risk. After I read each statement please tell me if you have thought similarly in the past week on a scale of 1 to 4. 1 being not at all, 2 being rarely, 3 being sometimes, or 4 being often. Do you have any questions before we begin?**

|  | **Not at All** | **Rarely** | **Sometimes** | **Often** |
| --- | --- | --- | --- | --- |
|  |  | | | |
| 1. I thought about my cancer risk when I didn’t mean to. | 1 | 2 | 3 | 4 |
| 2 I avoided letting myself get upset when I thought about my cancer risk or was reminded of it. | 1 | 2 | 3 | 4 |
| 3. I tried to remove my cancer risk from memory. | 1 | 2 | 3 | 4 |
| 4. I had trouble falling asleep or staying asleep because of pictures or thoughts about cancer that came into my mind. | 1 | 2 | 3 | 4 |
| 5. I had waves of strong feelings about my cancer risk | 1 | 2 | 3 | 4 |
| 6. I had dreams about cancer risk. | 1 | 2 | 3 | 4 |
|  |  | | | |
| 7. I stayed away from reminders of my cancer risk. | 1 | 2 | 3 | 4 |
| 8. I felt as if it hadn’t happened or wasn’t real. | 1 | 2 | 3 | 4 |
| 9. I tried not to talk about my cancer risk | 1 | 2 | 3 | 4 |
| 10. Pictures about cancer popped into my mind. | 1 | 2 | 3 | 4 |
| 11. Other things kept making me think about my cancer risk. | 1 | 2 | 3 | 4 |
| 12. I was aware I still had a lot of feelings about it, but I didn’t deal with them. | 1 | 2 | 3 | 4 |
|  |  | | | |
| 13. I tried not to think about my cancer risk. | 1 | 2 | 3 | 4 |
| 14. Any reminder brought back feelings about my cancer risk. | 1 | 2 | 3 | 4 |
| 15. My feelings about my cancer risk were kind of numb. | 1 | 2 | 3 | 4 |

**That is the end of that section. Thank you for your responses.**

**Have you discussed your results with any of your family members?**

**If yes,**

1. Who have you talked to about your positive result?
   1. Kids, Spouse, Siblings, Cousins, etc…
   2. How did they respond?
2. What did you talk about?
   1. Did you talk to them about getting tested?
      1. Do they want to get tested?
      2. Are there reasons why the people you talked to do not want to get tested?
      3. Are they aware that your first-degree relatives can get testing through Color for $50?
3. Are you concerned about your family members’ genetic cancer risk?

**If no,**

1. Is there any particular reason why you are hesitant to share this information with them?
   1. Not comfortable discussing my health information with relatives
   2. Genetic information is private and personal
   3. They would not find this information important
   4. You are not close enough to them
   5. Could not contact them

**Did you seek out support or community after receiving your result?**

**If so, what kind? From whom?**

Prompts:

- Family or friends?
- Community (ex: religious group)?
- Online support group?

**Next, I am going to read you a series of statements regarding the decision you made to enroll in the study and receive results from the genetic test. Please tell me how much you agree or disagree with each statement by choosing the response that best describes your feelings, from: Strongly disagree, Disagree somewhat, Uncertain, Agree somewhat, and Strongly agree.**

|  | Strongly disagree | Disagree somewhat | Uncertain | Agree somewhat | Agree strongly |
| --- | --- | --- | --- | --- | --- |
|  |  | | | | |
| 1. It was the right decision | 1 | 2 | 3 | 4 | 5 |
| 2. I regret the choice that was made. | 1 | 2 | 3 | 4 | 5 |
| 3. I would go for the same choice if I had to do it over again | 1 | 2 | 3 | 4 | 5 |
| 4 The choice did me a lot of harm | 1 | 2 | 3 | 4 | 5 |
| 5. The decision was a wise one | 1 | 2 | 3 | 4 | 5 |

**That is the end of that section. Thank you for your responses.**

# Previvorship and Self Concept

Doctors and researchers are always working on finding better ways of helping patients identify and manage cancer risk, to help them live longer and with the best possible quality of life. One of the ideas that people are talking about right now is the concept of “previvorship.”

You’ve heard of cancer survivors – people who have had cancer and been treated successfully, so that their cancer is either cured or controlled.

Previvors are individuals who have an increased risk for cancer but have not been diagnosed with the disease. This includes people who have a family history of cancer that defines them as high risk, carry a genetic variation that is known to cause cancer, or have other things going on that are known to greatly increase risk.

1. **What do you think of the term, “previvor”?**
2. **How would it feel to you if someone referred to you as a previvor?**

Identifying people as previvors might create the opportunity for peer support – for example, people who have a predisposition to colon cancer could connect with each other and share their knowledge, advice, and concerns.

On the other hand, some people might feel that the term “previvor” is an unhelpful label – one that could do more harm than good.

**What do you think?**

**As a part of this study, we developed individualized reports for each patient who tested positive for a genetic variant; we call them Previvor Reports.**

**Have you seen your Previvor Report?**

If yes,

1. What did you think of it?
   1. Did you appreciate having the Previvor report in addition to your Color results?
2. Did you find it useful?
3. Was it helpful when having discussions about your test results with your provider(s) or family?

If no,

1. Do you think having a report that details your results, general preventative care recommendations, and available resources would be useful to you?
   1. Why or why not?

# Economic Aims

**Instructions:**

Lastly, I will ask you a brief series of questions about the amount of time you spent completing follow-up activities associated with receiving your genetic testing results. In response to each question, please select the time period that most closely aligns with the amount of time you spent completing a given activity. The options are A) less than 30 minutes, B) 30 minutes to an hour, C) 1 to 2 hours, D) 3 to 5 hours, E) more than 5 hours.

|  | Less than 30 Minutes | 30 Minutes-1 Hour | 1 Hour- 2 Hours | 3 Hours- 5 Hours | More than 5 hours | |
| --- | --- | --- | --- | --- | --- | --- |
|  |  | | | | |  |
| 1. How much time did you spend on Color Health’s website reviewing your results | A | B | C | D | E |  |
| 2. How much time did you spend talking to a genetic councilor about your results | A | B | C | D | E |  |
| 3. How much time did you spend talking to your provider about your results | A | B | C | D | E |  |
| 4. How much time did you spend talking to family members about your results? | A | B | C | D | E |  |
| 5. How much time did you spend reviewing your Previvor Report? | A | B | C | D | E |  |

**Thank you, that is the end of this section.**

**What else would you like to share about your experience with cancer genetic testing?**

**Participant Demographics**

| **How would you describe your race? *(allow multiple responses)*** | - **American Indian/Alaska Native** - **Asian** - **Native Hawaiian or Other Pacific Islander** - **Black or African American** - **White** - **More than one race** |
| --- | --- |
| **Are you Hispanic or Latino?** | - **Yes** - **No** |

**Thank you so much for taking the time to speak with me**.

*Describe the process for receiving and redeeming the gift card.*

*Ask the patient to inform study staff if they have a change in contact information*

**END OF INTERVIEW.**

**Supplemental Material 5**. COREQ (COnsolidated criteria for REporting Qualitative research) Checklist

| **Topic** | **Item No.** | **Guide Questions/Description** | **Reported on**  **Page No.** |
| --- | --- | --- | --- |
| **Domain 1: Research team**  **and reﬂexivity** | | | |
| *Personal characteristics* | | | |
| Interviewer/facilitator | 1 | Which author/s conducted the interview or focus group? | 6 |
| Credentials | 2 | What were the researcher’s credentials? E.g. PhD, MD | 1 |
| Occupation | 3 | What was their occupation at the time of the study? | 6 |
| Gender | 4 | Was the researcher male or female? | 6 |
| Experience and training | 5 | What experience or training did the researcher have? | 6 |
| *Relationship with*  *participants* | | | |
| Relationship established | 6 | Was a relationship established prior to study commencement? | 6 |
| Participant knowledge of  the interviewer | 7 | What did the participants know about the researcher? e.g. personal  goals, reasons for doing the research |  |
|  |  |  | 6 |
|  |  |  |  |
| Interviewer characteristics | 8 | What characteristics were reported about the inter viewer/facilitator?  e.g. Bias, assumptions, reasons and interests in the research topic |  |
|  |  |  | 6 |
|  |  |  |  |
| **Domain 2: Study design** | | | |
| *Theoretical framework* | | | |
| Methodological orientation and Theory | 9 | What methodological orientation was stated to underpin the study? e.g. grounded theory, discourse analysis, ethnography, phenomenology,  content analysis |  |
|  |  |  | 7 |
|  |  |  |  |
| *Participant selection* | | | |
| Sampling | 10 | How were participants selected? e.g. purposive, convenience,  consecutive, snowball |  |
|  |  |  | 6 |
|  |  |  |  |
| Method of approach | 11 | How were participants approached? e.g. face-to-face, telephone, mail,  email |  |
|  |  |  | 6 |
|  |  |  |  |
| Sample size | 12 | How many participants were in the study? | 6,8 |
| Non-participation | 13 | How many people refused to participate or dropped out? Reasons? | 6,8 |
| *Setting* | | | |
| Setting of data collection | 14 | Where was the data collected? e.g. home, clinic, workplace | 6 |
| Presence of non-  participants | 15 | Was anyone else present besides the participants and researchers? |  |
|  |  |  | 6 |
|  |  |  |  |
| Description of sample | 16 | What are the important characteristics of the sample? e.g. demographic  data, date |  |
|  |  |  | 8 |
|  |  |  |  |
| *Data collection* | | | |
| Interview guide | 17 | Were questions, prompts, guides provided by the authors? Was it pilot  tested? | 6 |
|  |  |  |  |
| Repeat interviews | 18 | Were repeat inter views carried out? If yes, how many? | 6 |
| Audio/visual recording | 19 | Did the research use audio or visual recording to collect the data? | 6 |
| Field notes | 20 | Were ﬁeld notes made during and/or after the inter view or focus group? | 7 |
| Duration | 21 | What was the duration of the inter views or focus group? | 6 |
| Data saturation | 22 | Was data saturation discussed? | 6 |
| Transcripts returned | 23 | Were transcripts returned to participants for comment and/or correction? | 7 |

| **Topic** | **Item No.** | **Guide Questions/Description** | **Reported on**  **Page No.** |
| --- | --- | --- | --- |
| **Domain 3: analysis and**  **ﬁndings** | | | |
| *Data analysis* | | | |
| Number of data coders | 24 | How many data coders coded the data? | 7 |
| Description of the coding  tree | 25 | Did authors provide a description of the coding tree? |  |
|  |  |  | 7 |
|  |  |  |  |
| Derivation of themes | 26 | Were themes identiﬁed in advance or derived from the data? | 7 |
| Software | 27 | What software, if applicable, was used to manage the data? | 7 |
| Participant checking | 28 | Did participants provide feedback on the ﬁndings? | 7 |
| *Reporting* | | | |
| Quotations presented | 29 | Were participant quotations presented to illustrate the themes/ﬁndings?  Was each quotation identiﬁed? e.g. participant number |  |
|  |  |  | 8-14 |
|  |  |  |  |
| Data and ﬁndings consistent | 30 | Was there consistency between the data presented and the ﬁndings? | 8-14 |
| Clarity of major themes | 31 | Were major themes clearly presented in the ﬁndings? | 8-14 |
| Clarity of minor themes | 32 | Is there a description of diverse cases or discussion of minor themes? | 8-14 |

Developed from: Tong A, Sainsbury P, Craig J. Consolidated criteria for reporting qualitative research (COREQ): a 32-item checklist for interviews and focus groups. *International Journal for Quality in Health Care*. 2007. Volume 19, Number 6: pp. 349 – 357

**Supplemental Material 6.** Provider training topics

Content focused on guiding providers through the process of identifying and following up with high-risk patients. Topics will include identifying familial risk factors for cancer; deciding when genetic testing is appropriate; how to order genetic tests; how to interpret and speak to patient about results of testing; equipping primary care providers with the tools needed to incorporate actionable results into a patient’s health management plan, as well as the legal & ethical landscape around genetics.
